# Supplementary material for: A simplified approach to failure analysis of ball bearings combining Principal Component Analysis and Fast Fourier Transform
Source: PLoS One. 2025 Nov 13;20(11):e0336163. doi: 10.1371/journal.pone.0336163 (PMC12614601; doi:10.1371/journal.pone.0336163)
Supplement: Attachment — Submitted filename: PONE-D-25-26658-Review comments.docx [file pone.0336163.s001.docx]

**Review of Manuscript PONE-D-25-26658**

The manuscript presents a method for ball bearing fault detection using PCA and FFT. It highlights the ineffectiveness of traditional time-frequency techniques in dealing with non-stationary and noisy signals, and proposes an advanced approach for reliable predictive maintenance. The integration of PCA and FFT is designed to identify fault indicators and extract frequencies and harmonics characteristic of faults.

However, here are still some comments in the manuscript that need to be improved as follows:

1. The authors claim the proposed method is novel. However, the novelty isn't clearly demonstrated. They should provide a more detailed discussion on how it differs from existing methods. For example, how does it compare to the work of other researchers who have used similar techniques for bearing fault detection?
2. The mathematical descriptions are too concise. For instance, equation (5) introduces the optimal linear transformation but lacks a clear explanation of its significance. The authors should explain in detail how this transformation contributes to the proposed method.
3. The results are presented in a somewhat disorganized manner. With figures like Figure 4 to Figure 11, the authors should focus on the most critical findings that support their conclusions. They could also discuss the implications of the results in real-world applications in more depth.
4. The experimental validation section lacks sufficient detail. The authors mention the use of data from Case Western Reserve University but don't specify the exact type of defects introduced or the methods used to simulate different speeds.

The manuscript presents a valuable contribution to the field of bearing fault detection. However, it requires significant revisions to enhance its clarity, detail, and impact. Addressing the issues outlined in the specific review comments would strengthen the manuscript and its suitability for publication in PLOS ONE. The authors should pay particular attention to the clarity of the mathematical descriptions, the detail and presentation of the experimental section and results, and the discussion of the innovation and practical implications of their method. Upon satisfactory revision, the manuscript could be considered for publication in *PLOS ONE*.
